# Advanced Antimicrobial and Anti-Infective Strategies to Manage Peri-Implant Infection: A Narrative Review

**DOI:** 10.3390/dj12050125

**Published:** 2024-05-06

**Authors:** Yihan Li, Cameron A. Stewart, Yoav Finer

**Affiliations:** 1 Faculty of Dentistry, University of Toronto, 124 Edward St., Toronto, ON M5G 1G6, Canada; yihanli.li@mail.utoronto.ca (Y.L.); cameron.stewart@mail.utoronto.ca (C.A.S.); 2 Institute of Biomedical Engineering, University of Toronto, 164 College St., Toronto, ON M5S 3E2, Canada

**Keywords:** antibiotic prophylaxis, antimicrobial, biofilm, surface modification, immune response

## Abstract

Despite reductions in bacterial infection and enhanced success rate, the widespread use of systemic antibiotic prophylaxis in implant dentistry is controversial. This use has contributed to the growing problem of antimicrobial resistance, along with creating significant health and economic burdens. The basic mechanisms that cause implant infection can be targeted by new prevention and treatment methods which can also lead to the reduction of systemic antibiotic exposure and its associated adverse effects. This review aims to summarize advanced biomaterial strategies applied to implant components based on anti-pathogenic mechanisms and immune balance mechanisms. It emphasizes that modifying the dental implant surface and regulating the early immune response are promising strategies, which may further prevent or slow the development of peri-implant infection, and subsequent failure.

## 1. Introduction

Dental implants have greatly enhanced oral rehabilitation capabilities, becoming a routine aspect of dental treatment [1]. Despite the high success rates of dental implants, the risk of postoperative bacterial infection at the surgical site prompts many dentists to prescribe prophylactic systemic antibiotics. Prophylactic antibiotic use may manage oral infection and marginally improve implant success rates. However, systemic antibiotics may cause undesirable side effects such as life-threatening allergic reactions and the emergence of bacterial antimicrobial resistance (AMR), leading to substantial health and economic burdens [2]. Official reports from the World Health Organization (WHO) on antimicrobial resistance argued that AMR could result in 10 million deaths annually by 2050 [3]. Recommendations to reduce overuse of antibiotics are being made [4]. Additionally, chronic peri-implant disease, which begins with bacterial infection, is among the most common and serious complications following dental implant placement, causing significant discomfort and pain to patients [5]. Consequently, the reduction of systemic antibiotic use and simultaneous reduction in post-implant-placement infections and/or chronic peri-implant infections have become important goals in the development of dental implant biomaterials and treatment alternatives.

**Figure 1:**
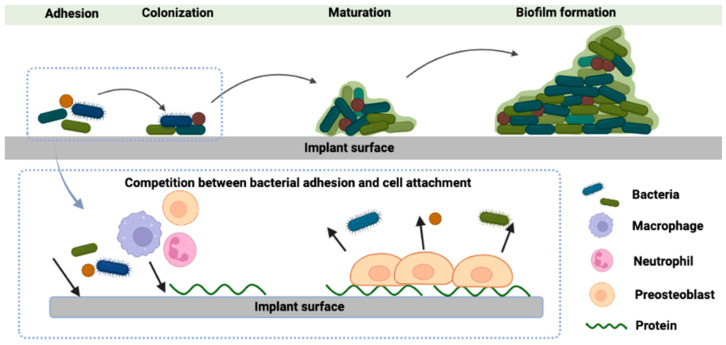
Schematic graph of biofilm formation on an implant surface. The process consists of four steps: (1) bacterial adhesion, (2) bacterial growth, (3) maturation, and (4) biofilm formation. Reproduced with permission from [13] and licensed under CC BY 4.0.

Bacterial infection of the implant, including implant abutment surfaces, involves complex interactions among bacteria, the implant surface, and immune response [6]. At first, the surface of the implant provides a foundation for bacteria to adhere to and form biofilms (Figure 1), facilitating subsequent microbial colonization on the implant surface. Once bacteria transition to their new sessile state, they establish microcolonies and produce protective biofilms, enabling them to survive in the challenging host environment. Meanwhile, bacterial invasion triggers a host immune response. In the long term, this inflammatory response may progress to peri-implant mucositis which is marked by inflammation confined to the epithelium, connective tissue loss, alterations in microvascular structures, and heightened infiltration of leukocytes [7,8]. The shift to peri-implantitis involves an increased influx of inflammatory cells into the affected region of the peri-implant mucosa, spreading the affected area to bone tissue [9,10]. Furthermore, a substantial presence of osteoclasts on the bone surface triggers bone resorption [11].

This narrative review summarizes the processes of implant surface biofilm formation and bacterial immune evasion; both are crucial elements in the development of peri-implant infection [12]. Subsequently, we review innovative antimicrobial and anti-infective biomaterial strategies designed to protect the wound site from bacterial infection at the boundary between oral tissue and the implant. Importantly, these strategies aim to achieve this goal while avoiding systemic antibiotic exposure and associated adverse effects.

## 2. Methods

### 2.1. Search Strategy

A comprehensive search of the most relevant literature was conducted for this narrative review. PubMed, Embase, Cochrane Library, and Google Scholar databases were thoroughly examined for articles investigating management of peri-implant infection. The search included articles published up to December 2023, resulting in a total of 175 references. Given that this review covers topics dating back to the 1950s, no time limits were set for the research, allowing for the selection of articles from that period up to today. The data compiled in this narrative review were acquired using keywords “bacterial adhesion”, “biofilm formation”, “antimicrobial”, “neutrophils”, “macrophages”, “T cells”, “immune evasion”, “immune modulation”, and “peri-implant infection”. Various combinations of these terms were utilized in the search process using Boolean operators AND and OR.

### 2.2. Study Selection, Inclusion and Exclusion Criteria

The relevant literature was acquired by examining the headings and abstracts of the chosen documents. Articles were based on their type and similarity in “Materials and Methods”. Our focus was primarily on recent publications investigating current approaches to managing post-operative and chronic peri-implant infection, including (1) antimicrobial strategies to inhibit implant surface bacterial adhesion and biofilm formation; and (2) anti-infective strategies to regulate the immune-inflammatory response. The study included systematic reviews, narrative reviews, clinical studies (case reports), in vitro studies and in vivo studies. Non-English-language articles and studies not specifically addressing the characteristics of dental implant surfaces were excluded from this review. Articles of interest referenced in reviews identified during the search were also examined. The inclusion criteria are listed in Table 1.

## 3. Molecular Mechanisms of Implant Colonization by Pathogens

### 3.1. Bacterial Adhesion and Biofilm Formation

The steps of bacterial adhesion can be divided into two stages: non-specific reversible attachment and specific irreversible attachment. On abiotic implant surfaces (bare non-living material surfaces), initial bacterial attachment primarily relies on non-specific forces like electrostatic forces [14,15]. On biotic implant surfaces (surfaces covered by living tissues), bacterial adhesion to extracellular matrix (ECM) molecules predominantly happens through specific binding of both piliated and non-piliated bacterial adhesins to host proteins. Following bacterial adhesion, bacteria adhere to one another and generate extracellular polymeric substances (EPSs) to form the biofilm matrix. Biofilm formation further contributes to the persistence of peri-implant infection by enhancing resistance to host immune responses, treatment, or mechanical removal and acts as the source of bacterial dissemination. Ultimately, biofilm dispersal occurs [16], potentially allowing bacteria to enter the bloodstream and cause systemic infection [17].

The complex mechanisms present during the formation and maturation of a biofilm on the implant surface present new potential targets for antimicrobial materials. Disruption of the ability of individual adhered cells to create a biofilm on the surface of the implant could prove to be an efficient method of reducing their pathogenicity and preventing implant infection-related diseases.

### 3.2. Immune Evasion

Various strategies enable bacteria to evade host immunity, including invading host cells, producing toxins, and modulating the immune response [6]. Consequently, enhancing our comprehension of osteoimmunology within the peri-implant environment might pave the way for the creation of novel therapeutic strategies that modulate bacterial interactions with the local immune response, maintain osseointegration, and prevent bone loss around implants.

Initially, bacteria can evade both antibiotics and host defenses by concealing themselves within host cells and bone tissues. The internalization of *S. aureus* into osteoblasts is facilitated by fibronectin, establishing a connection between staphylococcal fibronectin-binding protein (FnBP) and α5β1 integrin on osteoblasts [18]. This interaction prompts the upregulation of tumor necrosis factor-related apoptosis-inducing ligand (TRAIL), which subsequently activates caspase 8, leading to osteoblast apoptosis and consequent bone degradation [19]. Apart from infiltrating osteoblasts, *S. aureus* can also penetrate the canaliculi of live cortical bone. This evasion within bone tissue contributes to the stubborn nature of implant infections against host defenses and antibiotic treatments.

Moreover, other bacterial species can evade host immunity by combating host immune defenses with different mechanisms (Table 2): (1) Porphyromonas gingivalis (*P. gingivalis*), as the predominant pathogen in peri-implantitis development, disrupts with host immune response through a molecular mechanism. Its primary virulence factor, Gingipain R (Rgp), exhibits complement 5 invertase-like activity. This activity generates high concentrations of C5a ligand, regulating C5aR signal transfection in polymorphonuclear neutrophils (PMN). It interferes with the MyD88 signaling pathway and the mediated clearance of bacteria, and it inhibits host protective antibacterial pathways [20]. (2) Escherichia coli (*E. coli*) strains isolated from peri-implant infection sites display increased resistance to complement, which helps bacteria survive and reach the implant surface [21]. (3) *Actinobacillus actinomycetemcomitans* (Aa) is the only microorganism capable of producing leukotoxin (LTX) in the oral cavity. LTX, a pore-forming protein, targets the cell receptor lymphocyte function-associated receptor 1 (LFA-1), which is specifically expressed on leukocytes. The leukotoxin primarily induces damage to PMN, lymphocytes, and macrophages [22].

### 3.3. Modulation of Immune Response

**Figure 2:**
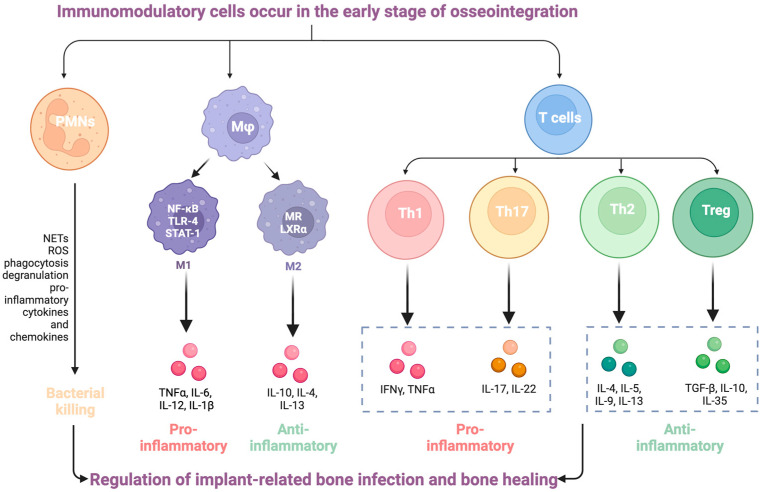
Evolution of immune response during biofilm formation and chronic development of implant-related bone infections. Reproduced with permission from [60] and licensed under CC BY 4.0.

Host immune responses not only react to bacterial contamination of an implant but also recognize the implant surface as a foreign body (Figure 2). Following biomaterial implantation, a multitude of circulating neutrophils and macrophages/monocytes migrate from the bloodstream to the implant surface and/or peri-implant tissue [23,24]. The accumulation of neutrophils in the tissue signifies the acute inflammatory response. As the primary immune surveillance arm of the innate immune system, neutrophils become activated within minutes and are the first responders to a biomaterial [25,26]. They play a crucial role in clearing cellular debris and pathogens through mechanisms like phagocytosis, reactive oxygen species (ROS) production, degranulation, and the formation of pathogen-encapsulating neutrophil extracellular traps (NETs) [27,28]. Moreover, neutrophils release various cytokines (i.e., interleukin (IL)-1β, IL-6, and IL-10) and chemokines (i.e., MCP-1 and CXCL1) to attract monocytes, thereby amplifying the inflammatory response [29]. Neutrophils are key players in combating infection around the implant, particularly those originating from Staphylococci [30,31]. The intracellular granules of neutrophils contain numerous potent antimicrobial proteins and components for generating high levels of ROS, rendering them highly effective in killing bacteria [32]. Reduced neutrophil function around the implant significantly increases the risk of biomaterial infection, emphasizing the importance of normal neutrophil function around the biomaterial [33,34].

Macrophages play a significant role in the initial inflammatory phase [35] and orchestrate the tissue microenvironment at the wound site [36]. They undergo polarization into two distinct phenotypes: the antimicrobial and proinflammatory M1-macrophages, and the anti-inflammatory and pro-regenerative M2-macrophages [37]. An imbalance in M1/M2 ratio, with a predominant M1 environment, can lead to chronic low-grade inflammation, osteolysis, loss of implant–bone integration, and implant loosening [38]. Conversely, regulating the M1/M2 balance of macrophages is crucial for wound healing, regeneration, and osseointegration [39]. Maintaining a balanced M1/M2 macrophage ratio is associated with M2-driven bone growth at the peri-implant site on the 10th post-implant day (PID) [40,41]. In the context of fracture healing, M2 macrophages contribute to both the resolution phase of inflammation and the recruitment of mesenchymal stem cells (MSCs) [42,43]. Additionally, M2 macrophages participate in the ossification phase of fracture repair [44,45]. Notably, a pro-regenerative M2 phenotype has the ability to produce trophic molecules, including Wnt ligands [46,47]. Elevated Wnt signaling has been linked to accelerated bone healing, enhanced implant osseointegration [48], and various functions during embryonic and organ development [49,50]. The regulation of local immune cell infiltration and macrophage polarization can impact both bone dynamics [51] and the progression of bone resorption [52], ectopic bone calcification [53] and solid tumor development [54]. Therefore, comprehending the diverse biological effects stemming from the intermediate stages of macrophage polarization remains an ongoing challenge.

Furthermore, the adaptative immune system’s role in tissue healing and regeneration appears significant [55]. Recent research indicates that reduced levels of pro-inflammatory cytokines produced by CD8+ T cells support new bone formation by MSCs and contribute to bone healing [56]. Additionally, CD4+CD25+FOXP3+ regulatory (Treg) cells are recognized as vital modulators of the immune response, capable of suppressing inflammatory response and facilitating reparative processes, thereby alleviating certain autoimmune diseases [39,40]. Interestingly, recent studies on the immune response to titanium implants revealed the activation of CD4+ T cells, while the activity of CD8+ T cells is suppressed. Hence, these findings suggest the presence of an adaptive immune response surrounding titanium implants [40,41].

The involvement of immune cells is pivotal in the activation of osteogenic pathways, strongly suggesting that early osseointegration involves immunomodulatory signals that mimic regenerative mechanisms [39,57,58,59]. The disruption of these pathways by bacterial infection and the resulting disruption of healthy implant healing further highlights the need for solutions that may prevent infection and also modulate immune activity.

## 4. Antimicrobial and Anti-Infective Strategies

### 4.1. Current Materials for Inhibiting Implant Surface Bacterial Adhesion and Biofilm Formation

Various properties of the implant surface can influence bacterial attachment, such as chemical composition, surface hydrophilicity, surface topography, and roughness. Irregular topography and rough surfaces provide an advantageous interface for bacterial colonization, offering protection against shear forces during initial reversible binding and biofilm formation. The chemical composition plays a crucial role in either promoting or inhibiting biofilm formation [61]. Utilizing surface modification techniques involving chemical species and altering surface topography to discourage microbial adhesion have the potential to decrease the prevalence and progression of peri-implant diseases [62,63]. These techniques for surface modification can be categorized into two approaches: those centered on physicochemical alterations to the surface and those involving the application of coatings containing antimicrobial agents.

#### 4.1.1. Physicochemical Surface Modification

Physicochemical surface modification techniques induce changes in the surface chemistry and nanostructure of the implant substrate, influencing interactions between the surface and cells by disrupting bacterial recognition of the surface and/or physically interfering in biofilm formation (Table 3). UV treatment has been found to improve the biocompatibility and antibacterial properties of implant surfaces. UV irradiation transforms titanium implant surfaces from a hydrophobic to superhydrophobic state, removing hydrocarbon contamination [64,65,66]. These significant alternations in surface properties have been shown to enhance osteoblast attachment and proliferation, leading to markedly improved osseointegration of titanium implants [67,68,69,70]. UV-treated surfaces exhibited a significant reduction in initial bacterial attachment and subsequent biofilm formation, although the overall viability of bacteria remains unaffected. However, further research is needed to ascertain whether this decrease in bacterial attachment and biofilm formation during implant placement can translate into enhanced long-term clinical outcomes for dental implants, particularly considering the temporary nature of the UV-treatment effect. Pesce et al. summarized that chair-side treatment of implants with UV does appear to be effective in improving osseointegration [71], but there was limited evidence that bacterial contamination of the implant surface was affected [71].

More recently, investigations have extensively explored biomimetic surfaces. Inspired by natural phenomena, several artificial nanotextured surfaces (NTSs) such as nanocones, nanofibers, and nanopillars were fabricated from various materials to attain bactericidal properties [72,73,74]. Bandara et al. suggested that damage to the bacterial membrane begins due to a combination of strong adhesion between nanopillars and the bacterium extracellular polymeric substance (EPS) layer along with shear forces exerted when immobilized bacterium attempt to move on the NTS [75]. However, it should be noted that these topographical changes may disappear over time due to surface wear on the implant neck during function.

#### 4.1.2. Implant Coatings

Titanium lacks inherent antibacterial activity [76] and poses a potential threat by allowing biofilm formation on implants [77]. Thus, various surface-coating techniques have been employed to alter the inherent surface characteristics of titanium implants (Table 3). These techniques include ion implantation [78], electrochemical anodization [79], ion exchange [80,81], sol-gel techniques [82], plasma spraying [83,84], and the inclusion of metal ions to regulate the initial adhesion of oral bacteria [85].

Recent advancements in nanotechnologies have sparked considerable interest in metal nanoparticles, due to their remarkable antimicrobial properties. These nanoparticles have the capability to impart antimicrobial properties on Ti implant surfaces without fundamentally altering their broader mechanical or physical properties, as seen with traditional coatings [86]. Silver is the most widely utilized antimicrobial metal ion [87,88,89,90,91,92,93,94,95,96,97], followed by zinc [98,99,100,101] and copper [102]. However, some studies have highlighted the antimicrobial properties of cerium [103], tantalum [104], titanium [87,105], and magnesium [101] nanoparticles. In these instances, much focus has been directed towards the synthesis of coatings containing ZnO, SiO_2_, Cu and Ag nanoparticles with biocidal effects [106,107,108,109]. The excellent antibacterial properties of these nanostructured agents are mainly due to their high ratio of surface area to volume, facilitating maximum contact with the environment and thereby enhancing reactivity [110]. Additionally, their small size facilitates easier penetration through cell membranes, directly influencing intracellular processes and intensifying reactivity and antimicrobial activity [111]. However, a significant drawback is that these particles usually lack inherent chemical linkages to the biomaterial matrix they are embedded in, leading to the release of these particles and other elutable materials over time. This leaching may lead to local and systemic health effects [112].

Utilizing anti-adhesive polymers to inhibit bacterial attachment is a popular strategy but creating implants with both antibacterial and osteogenic properties remains challenging [113]. Among these polymers, chitosan and carboxymethyl chitosan are widely employed for their antimicrobial properties. However, a notable disadvantage of these coatings is their non-specific suppression of osteoblasts and bacterial fixation. Consequently, functionalization with a peptide or a bioactive compound is necessary to enhance the adhesion of eukaryotic cells [114].

In summary, coating techniques have the potential to impart important positive characteristics directly on the dental implant surface, improving outcomes. Most experts agree that an effective coating method can significantly improve the mechanical and biological characteristics of dental implants [115,116,117,118,119,120,121,122,123]. Nevertheless, these methods present several limitations, such as a restricted long-term antimicrobial effect [124], insufficient adhesion of the coating to the substrate material [125], uneven thickness of the deposited layer [126], and disparities in the crystallinity and composition of the coating that compromise their effective integration with the bone [127].

#### 4.1.3. Ceramic Implant Biomaterials and Other Relevant Therapies

Ceramic materials have recently emerged as a popular alternative to titanium, largely driven by their superior aesthetic properties (Table 3) [128,129]. More importantly, ceramic materials have been associated with supposedly reduced microorganism adherence due to their surface roughness, surface free energy and surface chemistry [63,130]. Since zirconia has a lower affinity for bacterial attachment due to its lower surface free energy than titanium [131], it has the potential to replace the titanium in abutments not only for aesthetic reasons but also for biological reasons. However, there are still some concerns regarding the application of zirconia due to its comparatively lower mechanical strength, which can lead to higher rates of fracture and uncertain long-term survivability [132].

In addition to biomaterial antimicrobial strategies, clinical treatment options including use of chlorhexidine [133,134,135] and sterile saline [136], which might also prevent the development of peri-implant infections. Furthermore, the biomechanical design of implant abutment itself may contribute some positive effect. The presence of a micro gap in the implant abutment connection (IAC) serves as a site for the accumulation of dental plaque, promoting bacterial leakage that can lead to increased inflammatory cells at the IAC level, consequently causing peri-implant infection [137]. Research indicates that implant systems using an internal conical connection (ICC) are considered to offer better mechanical stability and seal performance [138], potentially making them a more favorable treatment option due to reduced bacterial microleakage and greater preservation of peri-implant bone tissue [139]. However, long-term follow-up studies are necessary to validate these results’ clinical significance.

**Table 3:** Current antimicrobial materials for inhibiting implant surface bacterial adhesion and biofilm formation.

Category	Material	Mechanism	References
Physicochemical surface modification	UV treatment	Decrease in initial bacterial attachment and subsequent biofilm formation	[64,65,66]
	Nanotextured surfaces (NTSs)	Bactericidal effect via a combination of strong adhesion and shear force due to topographical changes	[72,73,74]
Implant coatings	Metal nanoparticles	Antimicrobial properties	[87,88,89,90,91,92,93,94,95,96,97,98,99,100,101,102,103,104,105,106,107,108,109]
	Polymer—chitosan	Anti-adhesive and inhibit bacterial attachment	[114]
Ceramic material	Zirconia	Lower surface energy inhibiting bacterial attachment	[131]

### 4.2. Current Materials for Regulating the Immune–Inflammatory Response

As previously described, the interaction involving titanium dental implants, bone, and the immune system is intricate. Following implantation, a variety of host cells are recruited, engaging in interactions with the implant and each other. The physical and chemical characteristics of implant materials play a pivotal role in determining the extent of these immune responses during bone regeneration [140]. Dental implants may exhibit increasing levels of antigens (ions, nano and microparticles, and bacterial antigens) at the interface between the implant and tissue, triggering an immune–inflammatory response.

However, ongoing research on the immune response to implant-related bone infections reveals a discrepancy between robust pro-inflammatory immune reactions linked to osteoclastogenesis and bone deterioration and immune suppression that impairs effective eradication of bacteria. In light of this, below, we explore immunomodulatory strategies aiming to enhance and maintain long-term functional integration of dental implants in the human body (summarized in Table 4).

#### 4.2.1. Modulation of Neutrophils

After implant placement, neutrophils are conventionally regarded as basic components of the innate immune system, exhibiting a limited range of pro-inflammatory functions. Numerous studies have demonstrated that the surface properties of biomaterials can influence the activation of neutrophils [141]. Neutrophils respond differentially to alternations in the surface roughness and hydrophilicity of Ti implant surfaces (Table 4). Compared with smooth surfaces, rough surfaces demonstrate a more efficient induction of initial neutrophil adherence [142]. Ley et al. observed that rough regions on polymeric implants led to increased neutrophil death and ROS generation [143]. Abaricia et al.’s study revealed that neutrophils secrete higher levels of pro-inflammatory cytokines and enzymes on smooth or rough hydrophobic surfaces, along with enhanced neutrophil extracellular trap formation (NETosis), compared to rough hydrophilic surfaces [144]. This finding aligns with earlier research indicating that hydrophilicity significantly reduces the pro-inflammatory activation of leukocytes compared to hydrophobic and cationic surfaces [145,146,147]. Additionally, stiffness has been identified as a factor influencing neutrophil activation. Jefferson et al. discovered that higher stiffness substrates led to increased NET formation and higher secretion of proinflammatory cytokines and chemokines, and this effect was dependent on stiffness [148]. Oakes et al. also demonstrated that the area of neutrophil spreading increases with the rise in matrix stiffness, ranging from 5 kPa to 100 kPa [149]. These findings suggest that neutrophils regulate NET formation in response to physical and mechanical biomaterial cues, and this process may be regulated through integrin/FAK signaling [148].

Despite their often-overlooked role in the immune response to biomaterials, neutrophils play a crucial role in the initial inflammatory response to implant placement. Future research should focus on investigating the signaling pathways that mediate these responses and elucidate the role of NETosis in modulating the inflammatory response at the interface between biomaterial and tissue, with the aim of enhancing the efficacy of biomaterial implants [141].

#### 4.2.2. Modulation of Macrophage Polarization

Adjusting the innate immune reaction during the initial phases of the host reaction could be a preferable approach to enhancing implant integration and success. Macrophages are pivotal in the inflammatory process because of their cytokine production, influencing tissue healing and potentially contributing to implant failure [10]. As mentioned above, recent studies suggests that transient, early stage changes in macrophage polarization at the interface between tissue and implant, shifting from a pro-inflammatory (M1) to an anti-inflammatory (M2) phenotype, can alleviate the host inflammatory response to the foreign material and enhance downstream implant integration [150].

Numerous studies have shown that material modification with metal ions can positively modulate the polarization state of macrophages (Table 4). For instance, high levels of magnesium (Mg) on the implant surface decrease the secretion of pro-inflammatory cytokines, including TNF-a, IL-1b, IL-6, and PEG2 [151]. Costantino et al. illustrated the impact of Mg-based materials on macrophage-related-cellular activity at the molecular level [152]. Although a direct anti-inflammatory effect of Mg was not explicitly observed, there was an increase in both M1 and even higher M2-related cytokine production. Additionally, an additive modulating effect of Gd and Ag was noted, potentially working synergistically with Mg to promote the M2 macrophage phenotype [152]. Thus, implant surfaces treated with metal ions could modulate a pro-regenerative immune response in addition to their potential antimicrobial effects, thereby optimizing osseointegration.

Another strategy focuses on modulating the macrophage phenotype through polarizing cytokines (Table 4) [112]. Indeed, the inclusion of polarizing cytokines IL-4, IL-13, or IL-10 can stimulate macrophages to adopt the anti-inflammatory M2 phenotype [153]. Daniel et al. developed a nanometer-thick coating capable of releasing IL-4 from an implant surface, acting as a versatile cytokine delivery system to induce an early-stage shift in macrophage polarization at the tissue–implant interface [150]. Consequently, the addition of IL-4 triggers the polarization of macrophages from the pro-inflammatory (M1) to the tissue-regenerative (M2) phenotype, resulting in a pro-osteogenic response. This transition from M1 to M2 has been associated with an increase in bone anabolic factors.

Moreover, modifying surface properties may diminish the immune response to the implanted biomaterial (Table 4) [154]. For instance, it is established that modifying implant surfaces with titanium dioxide (TiO_2_) nanotubes positively affects osseointegration, possibly by shifting the phenotype of peri-implant macrophages from the pro-inflammatory (M1) subset to a pro-regenerative one (M2) [154,155,156,157,158]. The immunomodulatory ability to induce pro-regenerative macrophage polarization has also been investigated through additive manufacturing (AM) of porous titanium [159]. Additionally, the use of hydrophilic surfaces seems to stimulate macrophages to generate an anti-inflammatory microenvironment [160].

#### 4.2.3. The Role of T Cells

Although bacterial biofilm formation is considered a crucial initial stage in the progression of peri-implant disease, the immuno-inflammatory response triggered by the bacterial stimuli is responsible for the tissue damage associated with peri-implantitis [161]. Originally, the elicitation of the adaptive immune response was thought to be mediated by two subpopulations of effector CD4+ T cells: T helper 1 (Th1) and Th2 cells, distinguished solely by the cytokines they produced [162]. This “polarization model” was developed based on the type of the stimuli, wherein Th1 cells/cytokines were activated in response to certain bacterial or viral stimuli, while Th2 cells/cytokines were mainly associated with responses to helminthic infections [163]. This model has since been updated with the discovery of Th17 and Treg CD4+ helper T cells [164,165]. Treg cells produce the transforming growth factor (TGF)-b, IL-10, and IL-35 [166] and in many aspects counteract responses initiated by Th17 cells. Th17 cells, characterized by the production of IL-17, play various roles associated with the pro-inflammatory response, including recruiting neutrophils and macrophages [167], stimulating of pro-inflammatory cytokine synthesis, and generating antimicrobial peptides from immune and non-immune cells [168,169].

Titanium was reported to inhibit T-cell activation and the release of inflammatory cytokines [170]. However, the peri-implant mucosa was found to have an increased presence of Tolerogenic regulatory T cells (Tregs) compared with healthy gingiva in a mouse model [171]. Osteoimmunomodulation has increasingly been recognized as a crucial aspect of biomaterial-mediated bone formation [172,173]. The introduction of bone materials into the body prompts immune responses that play a role in determining the final outcome of the bone regeneration process (Table 4) [174]. Fei et al. proposed that bone-mimicking hydroxyapatite (HAp) nanorods with varying aspect ratios could regulate bone formation by modulating T cells and IL-22 during the bone regeneration process [174]. This finding sheds light on how nanomaterials can influence the immune response of T cells in osteogenesis and offers insights into designing biomaterials with osteoimmunomodulatory properties [173]. However, the precise role of T cells in defending against chronic implant-associated infections is not fully understood, and only a limited number of studies have explored this topic.

**Table 4:** Current materials used to regulate the immune–inflammatory response.

Category	Factor	Mechanism	References
Modulation of neutrophils	Surface roughness	Rough surfaces enhance initial neutrophil adherence more efficiently and increase neutrophil death and ROS generation	[142]
	Surface hydrophilicity	Hydrophilicity significantly reduces the pro-inflammatory activation of leukocytes	[145,146,147]
	Surface stiffness	The spread area of neutrophils increases with the rise in matrix stiffness	[149]
Modulation of macrophage	Metal ions (Mg, Gd, Ag)	Promoting the M2 macrophage phenotype	[151,152]
	polarizing cytokines (IL-4, IL-13, or IL-10)	Activating macrophages into the anti-inflammatory M2 phenotype	[153]
	Altering surface properties (nanotubes, AM porous titanium, hydrophilic surfaces)	Inducing pro-regenerative macrophage polarization	[154,155,156,157,158,159]
Modulation of T cells	HAp nanorods	Regulating osteogenesis by modulating T cells and IL-22 during the bone regeneration process	[174]

## 5. Conclusions

With the growing prevalence of implant placements, bacterial infection has become a heightened concern in implant therapy [60]. To reduce the risk of infection, antibiotic prophylaxis has been widely applied before implant placement. This may increase the risk of AMR development and generate substantial health and economic burdens. Therefore, we have summarized advancements in treatment strategies aiming to prevent or minimize post-implant-placement infections and implant loss and improve implant integration while reducing reliance on systemic antibiotics.

The primary risk of bacterial infection arises from bacterial evasion of the host immune response, with biofilm formation representing a significant mechanism for bacterial persistence. The presence of conventional implant materials fosters the formation of biofilm and prolongs infection. Therefore, there is a pressing need to tackle infections during the planktonic stage before they progress to biofilm formation and to prevent reinfection following antibiotic and surgical treatment. Novel therapeutic strategies, such as modification of the implant surface or modulation of immune cells, have been explored to reduce bacterial adhesion and inhibit biofilm formation while speeding up implant integration to reduce the window of infection susceptibility post-placement and modulating the immune response and its role in peri-implant disease. Nevertheless, disputes persist in the current research regarding the efficacy or practicality of the various methods and models developed. Furthermore, more in vivo studies are required to clarify the role and mechanism of each material parameter in the development of oral biofilm [61]. Long-term studies are also essential for evaluating the efficiency of biomaterials in alleviating chronic peri-implant infections, including peri-mucositis and peri-implantitis.

## 6. Future Perspective

Researchers should persist in the development of biocompatible peri-implant delivery systems, which can provide an antimicrobial effect directly to the wound site during healing while not eliciting AMR. Beyond traditional antimicrobial treatment, the immune modulatory approach presents a promising strategy for managing early bacterial infection. Immune modulation offers an additional medical treatment option, aiming to restore an efficient host response. It is expected that integrating antimicrobial treatment with immunotherapeutic intervention will facilitate successful management of implant infection in the future.

## Figures and Tables

**Table 1:** Inclusion and exclusion criteria.

Database	PubMed, Google Scholar, Embase, Cochrane Library
Publication date	Until December 2023
Keywords	“bacterial adhesion”, “biofilm formation”, “antimicrobial”, “neutrophils”, “macrophages”, “T cells”, “immune evasion”, “immune modulation”, and “peri-implant infection”
Language	English
Type of paper	In vitro studies, in vivo studies, clinical studies, reviews, systematic reviews
Inclusion criteria	Articles relating to main focuses with similar materials and methods
Exclusion criteria	(1) Non-English-language articles, books, other types of articles; (2) Studies not specifically addressing the characteristics of dental implant surfaces.
Journal category	All

**Table 2:** The mechanism of immune evasion by different bacteria species.

Method	Species	Mechanism	Reference
Invading host cells	*S. aureus*	Fibronectin	[18]
Combating host immune defences	*P. gingivalis*	Gingipain R (Rgp), immune response disruption	[20]
	*E. coli*	Increased resistance to complement	[21]
	*A. actinomycetemcomitans*	Leukotoxin (LTX), immune cell damage	[22]

## Data Availability

No new data were created in this study. Data sharing is not applicable to this article.
